# The Addict in Us all

**DOI:** 10.3389/fpsyt.2014.00139

**Published:** 2014-10-09

**Authors:** Brendan Dill, Richard Holton

**Affiliations:** ^1^Department of Linguistics and Philosophy, Massachusetts Institute of Technology, Cambridge, MA, USA; ^2^Department of Philosophy, University of Cambridge, Cambridge, UK

**Keywords:** addiction, self-control, desire, incentive salience, ego depletion, mindfulness meditation, mental contrasting, implementation intentions

## Abstract

In this paper, we contend that the psychology of addiction is similar to the psychology of ordinary, non-addictive temptation in important respects, and explore the ways in which these parallels can illuminate both addiction and ordinary action. The incentive salience account of addiction proposed by Robinson and Berridge (1–3) entails that addictive desires are not in their nature different from many of the desires had by non-addicts; what is different is rather the way that addictive desires are acquired, which in turn affects their strength. We examine these “incentive salience” desires, both in addicts and non-addicts, contrasting them with more cognitive desires. On this account, the self-control challenge faced by addicted agents is not different in kind from that faced by non-addicted agents – though the two may, of course, differ greatly in degree of difficulty. We explore a general model of self-control for both the addict and the non-addict, stressing that self-control may be employed at three different stages, and examining the ways in which it might be strengthened. This helps elucidate a general model of intentional action.

## Introduction

On a common conception, addicts and non-addicts are very different. Addicts’ compulsions drive them to act in ways that are quite foreign to the non-addicted. They consume drugs in the full knowledge that they are harmful, and in the face of a desire to stop, something that the normal agent does not do.

We argue here that this picture is quite misleading. Non-addicts, like addicts, have to contend with desires that are quite insensitive to their reflective judgments about what is good. And addicts, like non-addicts, have at their disposal a capacity for self-control that can enable them to resist and overcome these desires.

The situation faced by the addicted agent is thus parallel to that faced by the non-addicted agent. It is an extreme example of the same kind of thing. Both will have desires that persist even in the belief that their objects are worthless, or even actively harmful. And so both will be faced with the self-control problem of resisting these troublesome desires in the light of these beliefs. This self-control challenge, faced by both addicted and non-addicted agents, is the focus of this paper.

We begin by briefly outlining the empirical support for our first claim, that addictive desires are instances of a kind of desire common to all agents (see Desire). They result from a system – the “incentive salience” system – that has evolved to create desires, for foods and other things, that are independent from the agent’s evaluations of the worth of those things. What is different in the addict is not the intrinsic nature of these desires, but their origin. Addictive drugs cause the desire-formation process to malfunction, with the result that they come to be desired with an intensity and permanence that is quite out of proportion to any pleasure they have given. However, the same problematic features of addictive desires arise even when the incentive salience system does not malfunction. We see this in more mundane desires such as the craving for chocolate. We characterize the common features of these “incentive salience” desires, and contrast them with the more reasons-sensitive desires, which we call “cognitive desires,” on the basis of which agents reflectively deliberate about what to do. The competition between these two kinds of desire for control over behavior poses the problem with which we are concerned throughout the remainder of the paper: the problem of self-control.

We begin our discussion of self-control by arguing that an agent’s course of action is not solely determined by the relative strengths of her desires; it also matters whether, and how, she exerts *self-control* on behalf of some desires over others. Our argument centers on two subject populations whose behaviors are, we think, best explained as resulting from selective deficits in self-control capacity: subjects with lesions in the ventromedial prefrontal cortex (vmPFC), and subjects experiencing *ego depletion* (see The Existence of Self-Control).

The picture that emerges from these first two sections portrays intentional action as the result of a competition between two systems: the incentive salience system, which automatically guides behavior on the basis of appetitive desires, and the self-control system, by means of which an agent can, with effort, bring her actions in accordance with her more reflective desires. Though the conflict between these systems is typically more dramatic in addicts, it pervades ordinary action as well.

Though we offer some new arguments in its support, this two-system picture is far from novel. The basic outlines of the approach date back to Plato [(4), *Republic* Book IV] and the more contemporary version of this picture we present here has been defended before (5–8). What we hope to add to this literature is a more detailed picture of how these two systems interact to produce behavior (see Three Stages of Self-Control). We propose that there are three distinct loci of self-control conflict – at the point of deliberation, of formation of intention, and of execution of action – which we call the *deliberative, volitional*, and *implemental* stages of self-control. Distinguishing between these stages brings into focus the nature of the self-control challenge faced by addicts and non-addicted agents alike. Drawing on a large body of empirical work, we articulate the nature of the conflict between the self-control and incentive salience systems at each stage, and suggest ways in which each kind of self-control might be improved. What emerges is a single model of human motivational psychology that captures the predicaments of addiction and ordinary temptation with equal aptitude.^1^

## Desire

Let us start with the question of how we form desires. One might think – many have thought – that we are hedonists at heart. On such a view all of our desires stem from a fundamental intrinsic desire for pleasure. When we desire things other than pleasure we desire them *instrumentally*: that is, we desire them derivatively, because we believe that they will give us pleasure.

Many have objected that such an account makes us seem far too selfish: sometimes we want things because of the benefits that they will bring other people, independently of any benefits they may bring to us. We think that this point is probably right,^2^ but it is not our primary concern here. Our argument is rather that such a picture is wrong even when we consider such simple self-regarding desires as those we have for different foods. Suppose that an agent were to sample many different foods. Some they would like, others not, and they would then go on to regulate their future desires for them accordingly. We might expect these to be instrumental desires, formed in the service of the desire for pleasure. But the empirical evidence suggests not. It suggests instead that pleasure typically causes us to have intrinsic desires for the foods themselves, which then motivate independently of any beliefs about the pleasure that such foods will bring.

The crucial evidence for this is that our desires for different foods are not always directly responsive to our explicit beliefs about how pleasurable they are to eat. The desires do not need such beliefs to bring them into existence; and they can persist in their absence. We sometimes get a sense of this in our direct experience – many of us experience a desire to eat more of a thing (chocolates? over-rich desserts? peanuts? potato chips?) even when we know that we won’t enjoy it and that it may leave us feeling somewhat nauseated. However, the best evidence for this phenomenon comes from studies, not of normal foods, but of addictive drugs, and moreover, of how they work on rats. So let us start there, and then return to the case of how more normal foods work on us. Our account will follow the “incentive salience” theory developed by Robinson and Berridge (1–3).^3^

Addictive drugs artificially increase the levels of the neurotransmitter dopamine in the brain. Different drugs do this in different ways: nicotine stimulates the production of dopamine directly, opiates decrease the production of substances that inhibit the production of dopamine, cocaine reduces the activity of the system that reabsorbs dopamine after it has been released, and so on [(9), pp. 245–246]. What is remarkable is that these various substances with otherwise disparate biological and neurological effects have this single common feature: they all boost the effect of dopamine.

It is reasonable to infer that this shared neurobiological quirk must play a role in explaining these substances’ more obvious common feature: that they all cause addiction. Although there remains controversy here, this idea is borne out by the evidence. By boosting dopamine levels, addictive drugs artificially stimulate the mesolimbic dopamine system, which has long been known to play an important role in motivation. That is, they stimulate it directly, and not in the normal way via an experience that also gives rise to pleasure. (Compare getting someone to see stars by banging them on the head, rather than by showing them stars.) So to understand how drug addiction works, we need to understand what role dopamine plays in motivation.

For many years dopamine was thought to be a pleasure signal. But it is not. Whilst it is typically accompanied by pleasure, that is not what it is causing or registering [for a detailed defense of this claim, see Ref. (10)]. Separate the indicators of a rat’s pleasure (its facial movements) from the indicators of its desire (the effort it will expend to attain the thing), and you find that dopamine is linked to desire and not to pleasure. Artificially increase a rat’s dopamine levels by giving it amphetamines, and it will work much harder to get something even if that thing gives it no pleasure, and it knows it (11). Reduce the rat’s dopamine levels via genetic modification and it will fail to work for a thing even if that thing will give it great pleasure, and it knows it (12). Moreover – and this is crucial given the implications for addiction – if you increase the dopamine levels when a rat is sampling a foodstuff, what you bring about is not just an immediate desire for that foodstuff, but also a long-term dispositional desire for it (13). Show the rat the foodstuff again later, and it will still want it strongly.

What is happening here? Rats are opportunistic creatures, who need to be able to accommodate their tastes to a new environment. It makes sense for them to be able to regulate their desires in proportion to the pleasure that they get from various foodstuffs. Dopamine is clearly involved in this process. But it looks as though dopamine works directly on desires, without the need for the involvement of pleasure or beliefs about pleasure. It may be that dopamine release is typically *caused* by pleasure: in the case of most non-addictive foodstuffs, the most pleasurable ones will give the greatest dopamine release. But if dopamine is artificially increased, as it is by addictive drugs, then this leads to the production of desire independently of pleasure.

In fact, given what we have said, we need to identify two roles that dopamine plays in the production of desire. One, the *triggering role*, involves the triggering of occurrent desire: dopamine has a role in actually getting the rat to move toward the food in the moment. The other, the *formation role*, involves the formation of dispositional desire: dopamine works to set up a long-term disposition to want the food in the future.^4^ Stimulate a rat’s dopamine levels at the same time that it is consuming a certain food, and it will form a dispositional desire for that food (13). This is a focused desire: it is focused on the food that was being consumed when the dopamine was released. Present the food again, or present other cues that were associated with it, and the rat will want it, even if its dopamine levels are not then being stimulated. Dopamine thus creates a dispositional desire that, when cued by the relevant food or other associated cues, triggers an occurrent desire for that food.

The formation role that dopamine plays has often been described as a learning role. But that is misleading, since, at least within the more cognitive models that now dominate psychology, learning is best taken to involve a change in belief.^5^ It is not that the rat comes to believe that the food is going to bring it some advantage, and so forms an instrumental desire conditional on that belief. Rather, what is happening is that an *intrinsic* long-term desire for the substance is being created. If the desire is not reinforced, it will fade in time. But with desires put in place by addictive substances, this can take a very long time indeed – they may last for much of a rat’s life.

On the basis of this evidence, Robinson and Berridge (1–3) posited a motivational system, the “incentive salience” system, which has the following features. The incentive salience system creates dispositional desires for objects on the basis of those objects’ past association with reward. These dispositional desires, which we will call *incentive salience desires*, are activated – become occurrent – when the rat encounters the desired thing, or cues that have been associated with it. Once an incentive salience desire is active, it leads automatically to behavior in pursuit of the desired object. Crucially, the neural reward signal on the basis of which the incentive salience system acquires its desires is a dopamine signal. Thus addictive substances, by artificially boosting dopamine levels in the brain, produce a disproportionately large reward signal, which in turn causes the formation of a disproportionately strong incentive salience desire for the substance.

We have good reason to think this incentive salience system is present in humans as well as rats. The argument for this claim is an inference to the best explanation: the puzzling features of human addiction are best explained by the hypothesis that addictive desires are incentive salience desires. It explains the craving that is typically prompted by cues associated with the drugs: because of the artificial dopamine boost addictive drugs provide, subjects who consume these drugs acquire a long-term intrinsic desire for them, which is then triggered by the drug-associated cues. This account explains relapse, even after withdrawal: for the dispositional desire remains, ready to be triggered by the relevant cues.^6^ Finally, in human subjects, the account explains why the desires for drugs are so horribly independent from beliefs about their worth. For the incentive salience system is working quite independently of belief. The addict can know perfectly well that continued consumption would destroy everything that they hold dear. That does nothing to stop the rush of desire that is triggered by the thought or sight of the drug, or, more broadly, of the people, places or paraphernalia that have surrounded its consumption.

In addiction, the process whereby incentive salience desires are acquired malfunctions. When the system is functioning normally, the dopamine signal is proportional to the reward that the subject is experiencing, and thus the desire it produces is similarly proportionate. When a subject consumes an addictive substance, however, the artificial boost in dopamine that results can sever this link between “wanting” and “liking, ” leading to a desire for the substance that is way out of proportion with the pleasure it brings.^7^

But of course, much of what we have said about the incentive salience system still holds when it is not malfunctioning in this way. When it works well it still lays down long-term dispositional desires for things that have previously given pleasure; and these desires will be triggered by the relevant cues. If the things fail to give pleasure, then in time, the desire will diminish, though it will not evaporate straightaway. And if the thing continues to give pleasure, then the desire will be reinforced, even if the agent comes to believe that it is harmful.

To see this, consider the case of sugar. As far as we know, sugar has no direct effect on the dopamine system: it does not imitate dopamine, or inhibit re-uptake, or do any of the things that addictive drugs do. Nevertheless, rats that have been exposed to a sugar solution are strongly motivated to get it, just as they are motivated to get addictive drugs. In fact, if they have a choice between cocaine and sugar, around 90% of rats will take the sugar (14). It is possible that there is something special about sugar that causes the formation of long-term dispositional desires in this way. But it is equally possible that sugar is simply highly pleasurable.^8^ Certainly there is no reason to think that the rats’ desire-formation systems are somehow malfunctioning when they develop desires for foods that are rich in it.

Nor is there reason to think that things are any different for human beings. It has become commonplace to speak of sugar addiction; it is true that many subjects’ desires for sugar have a great deal in common with addicts’ desires for drugs. They too manifest in cravings that are highly cue-dependent, that are very powerful, and that persist in the face of the conviction that it would be better to eat less sugar. As with the consumption of sugar, so with many other pleasurable behaviors. Gambling, sex, surfing the web, watching daytime television – all of these have been alleged to give rise to addiction.

But we need to distinguish two things here: the “hijacking” of the desire-formation process that occurs with addictive drugs; and the nature of incentive salience desires themselves. The first of these features is unique to drugs: only substances that lead to artificial dopamine stimulation will hijack the desire-formation process in this way. We have no reason to think that sugar “addiction” results from a hijacking: there is no evidence that sugar leads to artificial boosts in dopamine. It is even more obvious that web-surfing and gambling do not stimulate dopamine in this way (since they are not ingested). So in none of these cases is there reason to think that the dopamine system has malfunctioned. Yet in every case there is reason to think that the motives to engage in these behaviors are insulated from the agent’s beliefs about what would be good. Incentive salience desires have this feature regardless of how they are acquired. It is exactly this feature that leads to the talk of addiction, since it is what substance and non-substance “addictions” have in common. Agents genuinely want to stop; and yet still they feel the pull of the desire.

We are therefore faced with a terminological choice: do we reserve the term “addiction” for desires formed by means of the dopamine hijacking process, and so say that sugar and gambling addictions are not addictions proper? Or do we use the term “addiction” to refer more generally to the predicament an agent faces when she has sufficiently strong and uncontrolled incentive salience desires, whatever their origin – and thus say that sugar and gambling addictions can be genuine addictions after all? Of course in a sense nothing hinges on the choice: once we are clear on the phenomena, it should not matter how we use the words. Nevertheless, talk of addiction brings with it so many expectations that in practical terms the choice matters deeply. We are torn on this question: RH is inclined to take the first option; BD leans toward the second. In the rest of the paper, we will side with BD and take the more inclusive definition, whilst saying nothing about the difficult question of when a “normal” desire for gambling or sucrose should be seen as an addiction.

What it is important to realize is that there is a contrast between these incentive salience desires, however caused, and many of our other desires. While incentive salience desires are by nature insensitive to our judgments about what is good, not all desires share this feature. In many cases, a desire is bound up with a reason or a justification: to want something is to want it for some reason.^9^ As one’s confidence in the reason diminishes, so the desire diminishes. Suppose that one of your favorite companies is bringing out a new model of some device that you particularly like; moved by the advance publicity you start to develop a hankering for it. But now the reviews come out, and without fail they are dismissive. The thing is clunky, ill-conceived, badly engineered, a definite step backwards. Your desire withers. You do not need to resist or overcome it. Once your beliefs have changed so that you see no reason to continue, the desire is no longer there. We do not have to think that these reason-based desires are always instrumental, i.e., that we only have them in order to get some other thing. But they are bound up with their reasons in such a way that they do not have a life of their own: they cannot live on without them, unlike the incentive salience desires, which can. We will call such desires *cognitive desires*, since they are sensitive to our cognition in a way incentive salience desires are not.

We should also distinguish incentive salience desires from another class of motivational states, namely habits. These clearly often play a role in addiction: it is not for nothing that we speak of an addict’s “habit.” Like incentive salience desires they are cued by circumstance, and often result in behavior that the agent rejects. Yet in so far as we have a good behavioral grip on them – behaviors like thumb-sucking, nail-biting, hair-pulling, and muscle tics – they differ in one crucial respect. The most effective treatment for them is *habit reversal therapy*, which involves monitoring the habit, and then learning an alternative response (15).^10^ And it seems that the most important part of this is simply the monitoring [(16); see also Ref. (17)]. Habits work automatically, but once they are monitored, the agent can override them. In contrast, while incentive salience desires are sometimes combined with an automatic element (reaching unawares for a cigarette), becoming aware of that element is not enough to remove their force. If they are to be resisted, they need to be overcome.

Let us summarize this section so far. We have contended that there are at least two distinct kinds of desire at play in human motivation. First, there are incentive salience desires, which are formed for objects on the basis of their previous association with either rewarding experience (when the system is functioning well) or artificial dopamine stimulation (when the system is hijacked by addictive drugs). These desires form the motivational basis of addiction, but also play an ever-present role in non-addicted agents’ motivation, encompassing at least the sphere of motives we normally call “appetites” even when these are well-regulated (desires for food, drink, sex, and many other typically pleasurable stimuli). Crucially, incentive salience desires motivate independently from an agent’s reflective judgments about what is valuable or even pleasurable. This distinguishes incentive salience desires from a second kind of desires, cognitive desires, which are sensitive to and based upon an agent’s reflective beliefs about what is valuable; e.g., the desire to read a certain book or pursue a certain career.^11^

How do these two kinds of desire, and our habits, interact to produce intentional action? A simple model, traditional in both psychology and philosophy, sees the efficacy of desires as simply a function of their *strength* (or of their strength together with the subject’s belief in how likely they are to be realized). On such a model what an agent does is simply determined by what she most wants to do. Incentive salience desires and cognitive desires will fight it out on the basis of their strength, and the stronger desire will control behavior.

There is a great deal of empirical evidence that tells against such a model, evidence that suggests that action is not simply dictated by the strongest desire. In particular, agents are not passive spectators of the competition between their desires for domination over behavior. Rather, the agent herself plays a much more active role in determining which desire triumphs, employing self-control to resist some desires, and to act on others. What determines an agent’s behavior, then, is not merely how strong her desires are, but also whether and how she exerts self-control.

Self-control is hard work. In the case of addiction, self-control is standardly employed to try to restrain incentive salience desires in the light of cognitive desires. Of course this attempt may not succeed. The addict may be aware that she (cognitively) prefers keeping her job to taking drugs, and be aware that taking drugs will cause her to lose her job, on that basis judge that she ought not to use, and yet *still* succumb to her desire for the drug. As R. Jay Wallace puts the point: “even if one succeeds, in the face of [an addictive] desire, in reasoning correctly to the conclusion that it should not be acted on, its continued presence and urgency will make it comparatively difficult to choose to comply with the deliberated verdict one has arrived at” (Ref. (18), p. 648). Moreover, even if one chooses to comply, it is hard work to convert that resolution into action.

Our contention here is that these points apply equally to *ordinary* action. For the features of addictive desire that pose self-control problems are features of incentive salience desires in general, and thus are shared by a wide range of non-addictive desires as well. Just as the motivational force of an addict’s incentive salience desire for heroin persists despite her judgment that she should not take it, the motivational force of an ordinary agent’s incentive salience desire for a cake will persist despite her judgment that she ought to have something more healthy instead. Whether the agent’s judgment or craving prevails is a matter of self-control.

We have already elucidated the essential features of the incentive salience system, and presented empirical evidence for its existence. However, we have so far said little about the nature of self-control, and have given no empirical argument for the existence of this phenomenon. We now turn to this task (see The Existence of Self-Control). Then we will be in a position to see how the different kinds of desires are mediated by the self-control system to produce intentional action (see Three Stages of Self-Control).

## The Existence of Self-Control

There are various reasons for believing in the existence of self-control as an independent system that is not reducible to strength of desire.^12^ Here, we present just one argument. The existence of a psychological system dedicated to a particular function is frequently accepted on the basis of evidence of a selective impairment in that function. For instance, autistic persons’ selective impairment in social cognition has been taken as strong evidence for the existence of a psychological system dedicated to social cognition (19), and prosopagnosic persons’ selective impairment in identifying faces has been taken as strong evidence for the existence of a perceptual system dedicated to face identification (20, 21). In general, a functionally specific impairment that shows up across multiple subjects seems best explained by positing the existence of a functionally specialized psychological system that is impaired or damaged in that subject population. Furthermore, by comparing these impaired subjects to healthy controls, we can uncover the causal–functional roles of the posited system.

Here, we follow this broad strategy, contending that the behavioral abnormalities of two different populations are best explained by a selective impairment in self-control: patients with lesions in the ventromedial prefrontal cortex (vmPFC), and (healthy) subjects who have undergone *ego depletion*. However, our claim here is more limited than those that have been made about social cognition or face recognition. We are not arguing that the system involved in self-control is *exclusively* dedicated to the task: that would require showing that *only* self-control is affected in these subjects, which is far from obvious (not least because we are not yet clear on what counts as an exercise of self-control and what does not). Our point is rather that the subjects in the two groups show a systematic loss of self-control even though there is no reason to think that their desires and beliefs have been affected; and hence that we have good reason for positing some kind of system that is responsible for self-control, whether or not that system is also responsible for other, unrelated processes as well.

Our pairing of vmPFC lesions and ego depletion may seem surprising, given that the two subject groups have been studied separately and in different subdisciplines (neuropsychology and social psychology). However, these two groups have an important common feature: they both behave as we would expect people to behave who are motivated overwhelmingly by incentive salience desires. This indicates that the motivational system that counteracts incentive salience desires’ effects on behavior is selectively impaired in these subject groups. As we will argue, these subjects’ deficits are best explained by appeal to the impairment of a psychological system that serves the function of governing behavior on the basis of cognitive desires. That is, these subjects seem to be suffering from selective impairment of the self-control system as we have described it.

This raises the question: how *should* we expect a person to behave who is motivated solely by incentive salience desires? We can make important predictions based on a single observation about how incentive salience desires are acquired: a dispositional incentive salience desire for an end state E is formed on the basis of past associations between E and a simultaneous dopamine reward signal (usually caused by pleasure, though sometimes caused by artificial dopamine stimulation, as with addictive drugs). The strength of a dispositional incentive salience desire for any end state E is proportional to the (recency-weighted) average of the past reward signals that have been associated with E (9).

Thus we can predict that incentive salience desires will only motivate agents to pursue ends that have been previously associated with co-occurrent reward. This means that agents will be unable to form incentive salience desires for ends that are not *immediately* rewarding, or not rewarding *to the agent*, since accomplishing these ends will not bring about a co-occurrent reward. This rules out two important kinds of ends. First, incentive salience desires will not motivate agents to pursue *long-term* goals, which produce valuable or rewarding consequences only long after their end states have been attained. Examples of such goals include the goal to pass an examination, the goal to lower one’s cholesterol, and (notably) the goal to quit an addictive drug: the benefits of achieving each of these goals accrue to the agent only long after the goal has been achieved. Second, incentive salience desires will not motivate agents to pursue *other-regarding* goals that, while they produce good consequences for others, are not immediately rewarding to the agent. Many moral and altruistic goals are likely to fall under this category: e.g., the goal to be honest when there is a prudential incentive to lie, the goal to avoid socially inappropriate or offensive behavior, and the goal to help others with whom one does not empathically identify.^13^ So we can predict that a person who is motivated solely by incentive salience desires will pursue predominantly *self-regarding* and *immediately rewarding* goals.^14^

Both vmPFC lesion patients and ego depleted subjects fit this prediction well. We will start with the vmPFC lesion patients, as their deficit is more dramatic.

Since Phineas Gage, the first recorded and most famous case of vmPFC lesioning, the two most salient features of vmPFC-lesioned patients have been their severe deficits in socially appropriate behavior and long-term planning (22). vmPFC lesion patients usually display “acquired sociopathy,” a disorder characterized by dampened and poorly regulated emotions as well as disturbed social decision-making. This typically causes vmPFC lesion patients, post-trauma, to be unable to maintain healthy social relationships or gainful employment (23, 24).

In addition to their sociopathic behavior, vmPFC lesion patients seem unable to base their behavior on the long-term consequences of their actions. The most famous demonstration of this deficit comes from the Iowa Gambling Task [IGT; (25)]. The IGT presents subjects with four decks of cards, which give differing monetary rewards when subjects draw from them. Two high-risk decks give large immediate rewards, but result in a long-term loss by giving even larger punishments; two low-risk decks present the long-term optimal option, yielding small but consistent rewards. Healthy control subjects will start by sampling all decks, temporarily favor the high-risk decks, and then learn to choose the low-risk decks after receiving punishment. vmPFC lesion patients, on the other hand, will continue to favor the high-risk decks throughout the task. The best explanation for this pattern seems to be that the vmPFC lesion patients are motivated by the short-term rewards offered by the high-risk decks, and cannot change their behavior on the basis of the cognitive desire to maximize their long-term payoff and the judgment that those decks have a suboptimal long-term predicted payoff.^15^

As has been noted since the first studies, however, vmPFC lesion patients typically display normal intelligence, intact knowledge of social norms, and the ability to make accurate predictions about future social and non-social consequences (26, 27). This indicates that these patients’ impairment is motivational rather than cognitive.

We submit that the best explanation for these results is that vmPFC lesion patients’ behavior is guided overwhelmingly by the incentive salience system, which activates self-regarding and short-term goals. This is why vmPFC lesion patients show deficits in the two otherwise unrelated domains of moral behavior and long-term goal pursuit: both kinds of behavior require the capacity to set and pursue goals to achieve end states that are not immediately associated with rewarding experience.^16^ However, these patients have normal explicit beliefs and evaluative judgments about what is good. So vmPFC lesion patients seem to be selectively impaired in their ability to act on their cognitive desires. This indicates that there is a psychological system, instantiated in or dependent upon the vmPFC, that (among other things) serves the function of controlling behavior on the basis of cognitive desires – i.e., the self-control system.

The self-control system can be impaired in healthy subjects as well, as is shown by studies on *ego depletion*. The ego depletion finding is that healthy (non-lesioned) subjects who exert self-control on one task will subsequently perform less well than control subjects on a second, unrelated task that also requires self-control (28). The large literature on ego depletion has demonstrated that many different kinds of task are ego depleting, from attention regulation (29) to making choices (30) to analytical thought (31). However, for our purposes, the most important ego depleting tasks are the motivational tasks, where subjects must exert self-control in order to override some desires in favor of others. On these tasks, ego depleted subjects show a similar pattern to vmPFC patients: they are selectively impaired in the pursuit of other-regarding and long-term goals.

Begin with other-regarding goals. The following results all support the claim that ego depleted subjects are less able to suppress selfish desires for the sake of other people:

- Ego depleted subjects are less likely to volunteer to help a victim of a tragedy (32).
- Ego depleted subjects are more likely to lie about their performance for monetary gain (33).
- Ego depleted subjects express more interest in sleeping with someone other than their romantic partner, are less able to suppress sexually inappropriate thoughts, and are more likely to inappropriately engage in sexual behavior with their dating partner in the laboratory when given an opportunity to do so (29).
- Ego depleted subjects are less effective at social self-presentation – for example, they are more likely to speak or disclose an inappropriate amount in conversation (34).
- Ego depleted subjects are more likely to respond destructively than constructively when their relationship partner behaves destructively (35).
- Ego depleted subjects are more likely to respond with aggression after an insult (36).

Moving on to long-term goals, the following results all support the claim that ego depleted subjects are less able to suppress short-term desires for the sake of long-term gain:

- Ego depleted subjects are less likely to choose to eat radishes rather than chocolates, or to restrain themselves from eating cookies when on a diet (28).
- Ego depleted subjects’ consumption of M&M’s candies is better predicted by their implicit evaluations of M&M’s than by their explicitly stated desires to eat healthy, while non-depleted control subjects’ consumption of M&M’s is better predicted by their explicit desires to diet than by their implicit evaluations (37).
- Ego depleted subjects are less likely to restrain themselves from drinking too much beer when they expect to take a driving test afterward (38).
- Ego depleted subjects are less likely to choose to study for a test rather than procrastinate by reading magazines or playing video games (30).
- Ego depleted subjects will drink less of a healthy but bad-tasting beverage (30).
- Ego depleted subjects are more likely to spend money impulsively when given the chance (39).

All these seem to be cases where the long-term value of a future outcome (e.g., health, sobriety in a driving test, achievement, savings) needs to override a craving to pursue some immediately rewarding end (cookies, chocolate, beer, video games, and impulse spending).

Like vmPFC patients, ego depleted subjects show a selective impairment that results in the relative domination of their behavior by incentive salience desires. Non-depleted subjects are better able to pursue long-term and other-regarding goals that cannot be activated by incentive salience desires. We think this data should be explained in the same way that we have explained the motivational deficits of vmPFC-lesioned patients. Healthy, non-depleted human agents are different from vmPFC lesion patients and ego depleted subjects in that they have a fully functioning self-control system, which is impaired or absent in these other populations. The self-control system enables healthy agents to override their incentive salience desires and control their behavior in accordance with their cognitive desires. This allows their motivational repertoire to include moral considerations, altruistic concern, and the long-term consequences of their actions. The powerful explanation of these two disparate bodies of data that we attain by positing the self-control system is, we submit, sufficient reason to accept its existence.^17^

Let us pause to address a worry regarding our argument’s appeal to the ego depletion findings.^18^ One might be wary of drawing any conclusions from the ego depletion findings, given the controversy that surrounds them. Given how hotly debated many of Baumeister and colleagues’ claims about ego depletion have been, is not it a bad idea to take those claims as premises in an argument? Though this concern is natural, closer examination reveals that the controversies surrounding ego depletion are orthogonal to our central claims.

First, there is an ongoing debate regarding the replicability of *one* of the empirical findings in the ego depletion literature. But this debate concerns not the central ego depletion finding itself, but a certain hypothesis about its physiological mechanism: Gailliot and Baumeister’s (40) claim that ego depletion is mediated by depletion of glucose in the bloodstream. Despite the original findings in support of this claim, more recent experiments have called it into question [e.g., Ref. (41, 42)]. However, our argument does not rely on this questionable finding. We only appeal to the ego depletion finding itself: the finding that subjects who exert self-control on one task perform less well than controls on subsequent self-control tasks. This finding has been replicated over 100 times, according to Inzlicht and Schmeichel (43). A recent meta-analysis of 83 studies reports that the ego depletion effect is both highly statistically significant (*p* < 0.001) and of medium-to-large size [Cohen’s *d* = 0.62; (44), p. 508]. Though there are still some skeptics [see Ref. (45)], the reliability and replicability of the ego depletion finding itself is widely accepted.

The other locus of controversy concerns what we call the *depletion question*: how does exerting self-control on one task impair self-control performance on subsequent tasks? Several answers to this question have been proposed. Most prominently, Roy Baumeister and colleagues have argued that self-control tasks all depend upon and use up a limited resource, which they call “willpower” (46). Their answer to the depletion question is simple: the first task uses up the willpower resource, leaving less willpower available than is necessary for optimum performance on the second task. However, this “resource theory” has recently been challenged by alternative accounts that claim we can explain ego depleted subjects’ impairment without appealing to a limited willpower resource. Several of these “anti-resource” accounts have been proposed (43, 47–49). Though the details of their accounts differ, these theorists all argue that exerting self-control decreases subjects’ *motivation* to exert further self-control – either by changing their beliefs or their desires – rather than by depleting a limited self-control resource.

It may seem that we need to take a stand on this controversy, siding with the resource theorist in claiming that there is a limited willpower resource that is depleted by self-control exertion. However, our argument does not require this claim. In fact, we think our argument is consistent with both the resource and anti-resource answers to the depletion question. To see how, we need to distinguish the depletion question from an alternative question one might ask about the ego depletion finding, which we call the *covariance question*.

The covariance question is: why does ego depletion affect the particular tasks that it does, and not others? In other words, the covariance question asks why the many abilities affected by ego depletion all stand or fall *together*. What do emotion regulation, making arbitrary choices, analytic thought, resisting tempting foods, altruistic behavior, and all the other ego depleting tasks have in common, so that ability on one of these tasks covaries with ability on all of the others? Why does not ego depletion make people worse at rote memory recall, instead of impairing analytic thought? Why does ego depletion make people more selfish, rather than making them more selfless? Why does ego depletion make people more impulsive, rather than making them more cautious? All of these questions fall under the umbrella of the covariance question.

A couple hypothetical scenarios show that the covariance question is dissociable from the depletion question. First, imagine that the ego depletion effect only occurred for a single task: say, the Stroop Task. The finding would be simply that subjects who perform the Stroop Task are subsequently impaired at further trials of the Stroop Task, but equally good at all other tasks. Here, there would still be an interesting depletion question: why does doing the Stroop Task temporarily impair subjects’ performance on the Stroop Task? But there would be no interesting covariance question: it is no mystery why ability on the Stroop Task covaries with ability on the Stroop Task. For the converse dissociation, imagine that instead of ego depletion, we had found an ego *augmentation* effect: that exerting self-control improved performance on subsequent self-control tasks. Clearly there is no depletion question to be had here, but rather an augmentation question, which would require a different kind of answer. If the same set of tasks were involved in ego augmentation as we have found to be involved in ego depletion, however, we would have the very same covariance question: why does performance on each of these tasks covary with performance on all of the others?

We are offering an answer to the covariance question. The best explanation for why the various abilities affected by ego depletion stand or fall together is, we propose, that they all depend upon the operation of the self-control system. To fully defend this claim, we would need to provide a theory of the causal-functional role of the self-control system, which showed how each of the tasks that is affected by ego depletion requires the self-control system, while each of the tasks that is unaffected by ego depletion does not depend upon this system. This task lies beyond the scope of this paper, though one of us (BD) hopes to undertake it in future work. Strictly speaking, our argument here defends an answer to only part of the covariance question: why does ability to act on the basis of long-term goals covary with ability to act on the basis of other-regarding goals? We have argued that the best answer to this question is that both of these abilities depend upon a system that serves the function of overriding an agent’s incentive salience desires to direct action on the basis of her cognitive desires. Whether this system is also employed in the other tasks affected by ego depletion is, as far as we have argued, an open question – though we are inclined to think that it is.

Our answer to the covariance question is consistent with any of the going answers to the depletion question. Clearly, it is consistent with the resource theory: on this view, all of the tasks affected by ego depletion depend upon the operation of the self-control system, which in turn depends upon a limited resource that is depleted by its operation. It is also compatible with the anti-resource theories. These theories explain depletion by appeal to a decrease in motivation *to exert self-control*. This raises the question: what is it to “exert self-control”? The most natural answer seems to be that to exert self-control is *to utilize one’s self-control system*. On the resulting anti-resource picture, depletion effects are explained by a decrease in subjects’ motivation to employ their system of self-control. This picture is consistent with our view as well.

So, our view is neutral on the debate between resource and anti-resource theorists about the mechanisms of ego depletion. Our argument for the existence of the self-control system does not rely on any premise that is at issue in this controversy. So, the fact that there is controversy about the depletion question cannot provide grounds for doubting the soundness of our argument.

We have argued that intentional action is the product of a competition between two different sorts of desires that is mediated by the self-control system. This thesis holds for both addicted and non-addicted agents. Both addicts and others have incentive salience desires, as we have already argued. These desires motivate *automatically*: as soon as an incentive salience desire is triggered, it drives an agent’s attention and behavior in pursuit of the desired object without conscious effort (even in spite of it). In addition, both addicts and others have cognitive desires: desires that are based in and responsive to the agent’s reflective judgments about what is good. In contrast with incentive salience desires, cognitive desires may include concerns for the long-term consequences of one’s actions, and the welfare of others as well as oneself. For better or worse, cognitive desires do *not* motivate behavior automatically. To guide her behavior on the basis of her cognitive desires, an agent must exert self-control. On the basis of data showing that some agents have a specific deficit in their ability to act on the basis of cognitive desires, we have argued that there is a system, the self-control system, dedicated to this task.

The self-control system serves primarily as the cognitive desires’ advocate within the brain. Since cognitive desires do not motivate automatically, it is up to the self-control system to make sure they are represented in the agent’s behavior. Whether an agent acts in accordance with her cognitive desires, in the face of a temptation to do otherwise, is not merely a matter of the strength of her cognitive desires, but rather a matter of her ability to exert self-control on their behalf. In other words, whether an agent’s cognitive desires triumph over her incentive salience desires depends on whether the self-control system manages to override the automatic influence of the incentive salience system. We will thus more often speak of the competition between *the self-control system* and the incentive salience system than of the competition between *the cognitive desires* and the incentive salience desires. But these are just two different ways of describing the same thing.

One might be tempted to explain addiction as the result of an impairment of the self-control system, but we think this idea is a non-starter. If addicts had an impaired self-control system, we would expect them to show behavioral impairments across the board: they would not only have trouble controlling their addictive desires, but would be self-regarding and focused on the short-term across all other domains as well. But this is clearly not the case: addiction does not lead to the domain-general deficits characteristic of vmPFC lesion patients and ego depleted subjects. Unlike vmPFC lesion patients, addicts do not act like sociopaths; unlike ego depleted subjects, addicts do not seem to be impaired in *all* tasks that require self-control, such as attention regulation or analytic thought. Moreover, given the right incentives addicts do succeed in controlling even their addictive desires.^19^

Instead, we propose that the primary difference between addicted and non-addicted agents lies in the very strong incentive salience desires possessed by the former. Whether caused by artificial stimulation of the dopamine system (as in the case of cocaine or amphetamines) or by the system working in the way in which it has evolved (as in the case of sucrose) the incentive salience desires involved in addiction are likely to be stronger than any of the incentive salience desires experienced by non-addicted agents. Thus it is a far greater challenge for addicts to override their incentive salience desires, due to the abnormal motivational force of their addictive desire. Though this self-control challenge is far more difficult for addicts than it is for others, the *structure* of the challenge is the same for both, as we will now try to show.

## Three Stages of Self-Control

We have claimed that intentional action results from the competitive interaction amongst desires mediated by the self-control system. This is to see self-control as in the business of regulating which of the subject’s desires gets to determine their behavior. But how does this work? Does self-control regulate which intentions the agent forms on the basis of their desires, or does it rather regulate whether they stick to their intentions? And might not it instead regulate which desires the agent has in the first place, or which judgments they form? We need to get clearer on what it is that self-control is controlling (or failing to control).

The philosophical literature on addiction presents several different, seemingly incompatible, answers to the question of where self-control breaks down in the case of addiction. Watson (50) and Levy (7, 51, 52) have both argued that addictive desires bias addicts’ *evaluative judgments* themselves, skewing deliberation so that they come to see taking the drug as the most attractive option. “One who is defeated by appetite is more like a collaborationist than an unsuccessful freedom fighter,” Watson declares colorfully [Ref. (50), p. 7]; and reiterates later: “We are not so much overpowered by brute force as seduced” (p. 10). Levy has developed this idea into a detailed account of addictive (and non-addictive) temptation, which he summarizes as follows:

In response to temptation the subjects spontaneously generate or retrieve from memory arguments in favor of weak-willed action. Since they lack the cognitive resources to reject these arguments, they experience judgment shift. They come to judge that the benefits of succumbing to temptation are higher than they previously had thought, or the costs of giving in are lower, or both, and they act accordingly [Ref. (51), p. 101].

On this account, self-control works to control one’s *judgments* in the face of the biasing influence of temptation.

In contrast, R. Jay Wallace argues that addictive desires make it difficult to *motivate* oneself to act on one’s evaluative judgments once they have been formed. He emphasizes this in the passage we quoted earlier: “Even if one succeeds, in the face of such a desire, in reasoning correctly to the conclusion that it should not be acted on, its continued presence and urgency will make it comparatively difficult to choose to comply with the deliberated verdict one has arrived at” [Ref. (18), p. 648]. On this account, self-control works to turn one’s judgments into a commitment to action: in other words, to form an *intention* to act.

Finally, Timothy Schroeder and Nomy Arpaly emphasize the power of *habits* in producing addictive behavior, observing that these automatic behavioral dispositions may place addicts who are trying to get sober in tempting situations, situations that tend to undercut their intentions:

The abstinent addict will do things without thinking about them at the time, only to find a difficult situation arising. “Why did I agree to go to that party where everyone will be using?” “Why did I turn down this street that leads me close to the dealers, and not down the next street?” “Why did I end up calling my old drug buddy when I was bored?” Questions like these are often answered by an addict’s unconscious behavioral tendencies [Ref. (53), p. 228].

On this account, self-control works even after one has formed an intention, to *implement* that intention in the face of the obstacles posed by one’s bad habits.^20^

Though each of these philosophers puts their favored locus of self-control conflict at center stage, we think there is no genuine disagreement between their claims. Instead, we favor a pluralist view: there are several distinct loci of self-control conflict. This view is advocated by Amelie Rorty in her classic article “Where Does the Akratic Break Take Place?” (54). Rorty begins by identifying several “stages on thought’s way to action,” and observes that “these distinctions allow us to locate the junctures where psychological akrasia can occur, in ways that explain the occurrence of behavioral akrasia” (334). These “junctures” at which self-control failure can occur are also the places where self-control might be improved: “the place where the akratic break takes place also locates the place where the self-reforming akrates can best intervene to remedy his condition” (334).

In this section, we follow Rorty’s strategy: first, distinguishing different stages by which thought leads to action; second, showing how self-control conflict arises at each of these stages; and third, showing how intervention at each of these stages can help an agent win the struggle to govern her own behavior.

We propose that there are at least three distinct stages by which thought leads to action, which we will call the *deliberative* stage, the *volitional* stage, and the *implemental* stage:

(1) In the deliberative stage, the agent forms a *judgment* as to what action is best. This is the locus of self-control conflict Watson and Levy identified: the deliberative challenge of coming to a clear-eyed evaluative judgment in spite of the biasing influence of incentive salience craving.
(2) In the volitional stage, the agent chooses an *intention* to pursue. This is the locus of self-control conflict Wallace identified: the volitional challenge of willing yourself to pursue the end you have already judged to be best.
(3) In the implemental stage, the agent selects *actions* that implement her chosen intention. This is the locus of self-control conflict Schroeder and Arpaly identified: since habits are brute, unmotivated behavioral dispositions, they can cause goal-discrepant behaviors even when one is fully committed to a goal pursuit. So, it is during the implemental stage that one must grapple with and overcome one’s habits.^21^^,^ ^22^

We now proceed to discuss these stages in detail, with the aim of showing how self-control at each stage works similarly in addicted and non-addicted agents alike. For each stage we then briefly outline the ways in which self-control might be improved, again for both addicts and non-addicts alike.

### Deliberative stage

#### Locus of deliberative self-control conflict: attention

As we have said (see The Existence of Self-Control), a central function of the self-control system is to control behavior on the basis of an agent’s all-things-considered judgments of the values of potential actions and their outcomes. But in order to do this, an agent must first form the evaluative judgments on the basis of which she aims to control her behavior. This involves creating mental simulations of various potential actions and their consequences, and then comparing them against one another on the basis of relevant evaluative criteria. This task of practical deliberation requires the agent to keep several different detailed simulations of actions in working memory simultaneously, attend to the evaluatively relevant features of each, and then compare them against one another. Since the capacity of working memory is limited, an agent will only be able to focus on a subset of the potentially relevant features of her different options. Thus what judgment she ultimately forms will depend to a large extent on what evaluatively relevant considerations capture her attention.

Consider, for instance, an alcoholic deliberating about whether to have another drink at a business dinner with a client. What choice she judges best will depend on what features of her options she attends to while deliberating. If she focuses exclusively on the features she finds attractive about the drink – the refreshing, pine-tree taste of a gin and tonic, the loose euphoria of inebriation – she will judge that having another drink is the thing to do. However, if she attends to the longer-term consequences of having another drink – the resulting drunkenness rendering her unable to comport herself appropriately in front of her client, her potentially losing business as a result, and the negative consequences of this for her professional reputation and career – she will likely judge that she ought to order a soda water instead. The judgment she makes about what is best to do will depend upon how she directs her attention during the process of deliberation.

The self-control and incentive salience systems will pull an agent’s attention in different directions as she deliberates. An active incentive salience desire pulls an agent’s attention to the attractive features of its object, thereby biasing the agent’s deliberation in its favor. Only by exerting self-control can an agent attend to the reasons not to act in accordance with her incentive salience desires – i.e., the long-term consequences of her actions for things she reflectively values. It is thus over the control of attention that the deliberative stage of the competition between self-control and incentive salience is waged.

#### Role of the incentive salience and self-control systems in deliberation

If we are correct that the self-control system is the system that is impaired by ego depletion, then we can infer its functions from the capacities that are impaired in ego depleted subjects. It is thus instructive that ego depleted subjects show impairments in both analytic thought (31, 55, 56) and selective attention (29, 31, 40). Since practical deliberation requires both selective attention and analytic thought, we should expect ego depleted subjects to be impaired in this capacity as well. This means that the self-control system not only serves the function of controlling behavior on the basis of evaluative judgments already made, but is also deployed in the formation of evaluative judgments themselves.

However, the self-control system does not have complete sovereignty over attention. An active incentive salience desire exerts powerful influence over attention, drawing it toward the desired object and its most attractive features. This involuntary attentional pull has a significant biasing effect on practical deliberation. By automatically directing an agent’s attention to the most attractive features of the desired object, an incentive salience desire can lead an agent to form evaluative judgments that give disproportionate weight to these features. This can lead agents subject to incentive salience cravings to form evaluative judgments that treat the desired object as much more valuable than they would judge it to be in the absence of craving.

This biasing effect has been demonstrated in empirical studies on both addicts and non-addicts alike. The most vivid display of this effect in non-addicts comes from a study in which the experimenters asked male subjects to answer survey questions while looking at pornography and masturbating (57). The sexually aroused subjects, when compared with non-aroused controls, reported being significantly more willing to engage in sexual behaviors they considered deviant (e.g., bisexual group sex) and to act immorally in order to have sex (e.g., slipping a woman a drug to get her to have sex). The influence of these subjects’ active sexual desire went beyond their overt behavior, biasing even their *judgments* about what it would be pleasurable or morally acceptable to do. Less dramatically, some studies have shown that occurrent cravings for food make people overestimate how much they will enjoy foods in the future [(58); see also (59)]. Hence why it is dangerous to go grocery shopping while hungry.

Addictive desires have the same kind of biasing influence on evaluative judgment as sexual desire and hunger, as demonstrated by Badger et al. (60). Badger et al. studied a set of heroin addicts undertaking rehabilitation treatment who were receiving daily a heroin substitute medication Buprenorphine (BUP) to alleviate withdrawal symptoms. The experimental task asked these subjects to choose between receiving different amounts of money and receiving an extra dose of BUP, to be administered five days later.^23^ The crucial manipulation was that one group of subjects was asked to make this choice while in a current state of craving, before they had received that day’s dose of BUP, while a second group of subjects was asked to make the same choices while satiated, immediately after receiving their dose of BUP. The satiated subjects placed a substantially lower dollar value on the extra dose of BUP ($35) than the craving subjects, who valued the extra dose almost twice as much ($60). Notice that the difference in value here is for a dose to be received 5 days later – so subjects had no reason to think their current state of craving would have any influence on their enjoyment of the extra dose. And yet the currently craving addicts still judged receiving an extra dose 5 days later to be a more valuable outcome than the satiated addicts did. This seems best explained by the attention-biasing effect of active incentive salience desires: by drawing the craving subjects’ attention to the attractive features of the extra BUP, their desire led them to judge it more valuable than they would have in the absence of craving.

#### How to improve deliberative self-control: mindfulness meditation

So, active incentive salience desires bias attention in both addicts and non-addicts, leading agents to disproportionately value the object of their current craving in their deliberative judgments about what is best. But agents can overcome this bias by exerting self-control, directing their own attention rather than letting it be guided by their current desire. This account yields a testable prediction: deliberative self-control can be aided by improving agents’ selective attention. In other words, the better an agent’s capacity to control her attention, the better she will be able to overcome the biasing influence of incentive salience-based temptation.

This prediction is confirmed by research on *mindfulness meditation*. Mindfulness is a traditional meditative practice that involves actively focusing one’s attention on some aspect of one’s present experience for an extended period of time. (Paradigmatically, one focuses on the experience of breathing.) Among the many psychological benefits of training in mindfulness meditation is an improvement in selective attention: both brief and long-term mindfulness training improve subjects’ ability to selectively control their attention, as measured by many classic tests of attention regulation (61–63). If our picture of deliberative self-control is correct, then these improvements in selective attention should help subjects to better resist incentive salience desires. And this is exactly what the data shows.

This prediction has been robustly confirmed in studies of addicts [for a review, see Ref. (64)]. Randomized and controlled studies testing a mindfulness training intervention for addiction have shown that mindfulness training leads to a significant reduction in use of the addictive substance and a significantly lower chance of relapse, both when compared to a no-treatment baseline (65, 66) and when compared to conventional addiction treatments (67–69). One study found that smokers high on dispositional mindfulness measures are less likely to relapse after quitting than smokers lower in dispositional mindfulness (70). Finally, at least two studies have found that addicts who undergo mindfulness training not only use the addictive substance less, but also experience less intense *cravings* for the substance (68, 71).

Mindfulness-based interventions help non-addicts to overcome incentive salience temptations as well. In particular, several studies have shown mindfulness training to help obese or overweight subjects to achieve their weight-loss goals [(72–74); see also (75, 76)]. In a recent review, O’Reilly et al. (77) found that 18 out of 21 reviewed studies of mindfulness-based interventions for obesity-related behaviors reported significant decreases in the targeted behaviors.

One study directly supports our hypothesis that the mechanism behind these successful interventions is an improvement in deliberative self-control (78). This study investigated the temporal discounting of food rewards in obese and healthy-weight individuals by offering them a choice between a large, delayed food reward and a small, immediate food reward. In an initial test, obese subjects showed a much steeper discounting curve than controls – that is, they were willing to give up a larger delayed reward for a smaller immediate reward. This is what we would expect, given that the obese subjects are experiencing a stronger incentive salience craving for food, which draws their attention disproportionately to the attractive features of the immediate reward. After the initial test, some of the obese subjects undertook a 50-min training session in mindful eating, while others just watched an educational video on nutrition. These subjects then completed the temporal discounting test again. Obese subjects who underwent mindfulness training subsequently showed a significantly less steep discounting curve than they had in the initial test: they were more willing than before to give up a smaller immediate reward for the sake of a larger delayed reward. (Subjects who watched the educational video showed no such improvement.) What this suggests is that the brief mindfulness training session helped the obese subjects to overcome the biasing effect of their food cravings and form more normal judgments about the relative values of immediate and delayed rewards. In other words, mindfulness training improved these subjects’ deliberative self-control.

We submit that our model of deliberative self-control provides the best explanation for the above results. An important first step in overcoming an active incentive salience desire is to form a clear-eyed evaluative judgment that indulging one’s craving will lead to worse consequences than refraining from doing so. An active incentive salience desire automatically biases one’s attention to the positive features of the object desired, leading agents to overestimate the value of satisfying their current desire. Mindfulness meditation training makes agents more skilled at self-controlled attention regulation, and thereby improves their ability to resist the biasing effect of active incentive salience desires on evaluative judgment. It is thus by improving agents’ capacities for deliberative self-control that mindfulness meditation helps addicts and non-addicts alike resist the influence of their incentive salience desires.

### Volitional stage

#### Locus of volitional self-control conflict: goals

The second stage of self-control is the volitional stage: after one judges what is best (deliberative stage), one must choose a goal to pursue (volitional stage) before one begins implementing that goal pursuit in one’s behavior (implemental stage). In other words, between judgment (deliberative) and action (implemental) lies *choice* (volitional), and to exert volitional self-control is to exert self-control in choosing a course of action. This was the self-control task identified by Wallace, of “choos[ing] to comply with the deliberated verdict one has arrived at” (648).

Some readers may be skeptical that the act of making a choice is really distinct from the act of forming an evaluative judgment. Our first response would be to note that the possibility of *akrasia*, choosing against one’s own best judgment, seems to require such a distinction. But, of course, people who are skeptical about the judgment/choice distinction will be skeptical about the existence of *akrasia* as well, and so this line of argument will seem to be begging the question.^24^

However, we think there is empirical evidence demonstrating that making a choice is psychologically distinct from forming an evaluative judgment. A study by Vohs et al. [(30), Study 6] shows that choosing to act on one’s evaluative judgments (volition) requires more self-control than merely forming evaluative judgments (deliberation). In this study, all subjects were presented with a webpage that gave various options for customizing a desktop computer for purchase. Some subjects were asked to choose between the customizations (the choice condition), while others were asked to consider the customization options and “form an opinion of the information, thinking about what [they] would prefer” (892), but importantly, were *not* asked to implement their judgments by selecting their preferred options on the website (the deliberation condition).

The dependent measure of this study was subjects’ subsequent persistence on an impossible anagram task, a task that has been shown to measure self-control capacity (28). What Vohs et al. found was that subjects in the choice condition, who had made a series of active choices, persisted significantly less on this task than subjects in the deliberation condition. This shows that the act of *choosing* involves an exertion of self-control that goes beyond the self-control required to form an evaluative judgment. These results not only dissociate choice from evaluative judgment, but also show that choice involves the exertion of self-control. In other words, this study establishes the existence of volitional self-control as a psychological task that is distinct from deliberation to a judgment.

So let us take as given the existence of volitional self-control and now ask what it involves. What is the psychological process involved in making a choice, and why might it require self-control?

We suggest that the exercise of choice involves the selection and activation of a kind of motivational mental state that psychologists call a “goal.” A *goal*, in the technical sense used by psychologists, is a mental representation of a desired end that directs behavior in pursuit of that end.^25^ We take it that such states often constitute *intentions*, as philosophers understand this term. The large research literature on goals, which we do not have the space to review here, has shown them to be a robust psychological natural kind with a distinctive suite of cognitive and behavioral signatures [for a review, see Ref. (79)]. Active goals direct attention, cognition, and behavior in a flexible and instrumentally rational way in order to bring about the end state that they represent. One primary way for a goal to be activated is simply for subjects to form a conscious, deliberate intention to pursue a certain end. We thus submit that volitional choice is best understood as the self-controlled act of activating a goal with a certain end.

#### Role of the incentive salience and self-control systems in volition

We have already seen how self-control plays a role in volition: Vohs et al.’s subjects had to exert self-control to go beyond forming a judgment and activate a goal to act in accordance with that judgment. Crucially for our purposes, however, self-controlled choice is not the *only* route by which goals can be activated. Goals are also activated automatically by incentive salience desires, as we shall now explain.

A series of experiments by Henk Aarts and Rudd Custers have demonstrated that a goal to pursue a certain end state can be non-consciously activated by subliminally associating positive affect with that end state (80–85). Aarts and Custers first demonstrated that subliminally associating positive affect with a goal caused subjects to report greater *wanting* to pursue the goal [(80), Study 1], and then showed in subsequent studies (cited above) that this greater wanting leads subjects to behave in the ways characteristic of goal activation. These results seem best explained by appeal to incentive salience desires. We have already seen (see Desire) that incentive salience desires are proportional in strength to the previous association of the desired object with reward, and are automatically activated by encounters with desire-associated stimuli. Thus we should expect that Aarts and Custers’ intervention to associate positive affect with an end state would activate an incentive salience desire to attain that end state. And as we would predict, this association leads subjects to *want* to attain the goal. This gives us good reason to think that Aarts and Custers have activated goals in their subjects *by means of* creating and triggering incentive salience desires. Thus their findings strongly indicate that an active incentive salience desire for an object automatically and non-consciously activates a *goal* to attain that object, which then directs behavior in pursuit of its attainment.

On reflection, this is exactly what we should expect. The incentive salience cravings that addicts feel for heroin or non-addicts feel for sugar or sex do not merely influence behavior by biasing deliberative judgment. These desires seem to have *direct* motivational power, *pushing* the addict to shoot up or the non-addict to bite into the cake before either has a chance to even consider whether this is a good idea. Incentive salience desires seem to directly guide behavior in the absence of counteractive self-control, and now we can see why: cravings activate *goals*, which automatically guide action toward the attainment of the thing that is craved.

Thus, the challenge of volitional self-control in the face of an active incentive salience desire is to resist the automatic activation of the goal to attain the desired object, and instead activate an alternative goal that accords with one’s deliberative judgments about what is best. Only one goal can guide behavior at a time; in fact, a dominant goal actively *suppresses* the accessibility of the most attractive alternative goals (86).^26^ Thus the self-control system and the incentive salience system can be seen as competing in a “horse race” of goal activation, where the winning system is the one whose favored goal is made most active and thereby comes to dominate downstream behavior. The stronger the incentive salience desire, the more activation it will give to its favored goal, and thus the greater exertion of self-control will be required to activate an alternative goal enough to override it. This is why restraining yourself from acting on an addictive desire is far more difficult than restraining yourself from eating a chocolate cake.

#### How to improve volitional self-control: mental contrasting

If volitional self-control is a matter of giving sufficient activation to one’s deliberatively chosen goal, then we should expect that any procedure that leads to greater activation of a consciously chosen goal will help agents to overcome temptation by incentive salience desires. The “mental contrasting” procedure, created and researched by Gabriele Oettingen, is an intervention of this kind. In this procedure, subjects who wish to attain a goal are asked to undertake two imaginative steps: first, imagine a “positive fantasy” of the goal’s being attained, and all the beneficial consequences that would follow goal attainment; second, *mentally contrast* this positive fantasy with the “negative reality” of one’s present distance from achieving the goal and the obstacles lying in the way of goal attainment. Several studies have shown that this mental contrasting procedure powerfully increases subjects’ motivation to attain the goal, causing them to expend much more effort in pursuit of the goal (87–91). What explains this effect?

We offer the following explanation. Goal pursuit research has independently shown that the activation level of a goal is automatically modulated based on three major factors: (a) *value*, the perceived value of achieving the goal (82, 92–94); (b) e*xpectancy*, the perceived probability of attaining the goal (93); and (c) *discrepancy*, the perceived effort required to attain the goal (95–97). Goal activation is strongest when expectancy, value, and discrepancy are all high.

We propose that the mental contrasting procedure activates goals by means of boosting value and discrepancy: the “positive fantasy” increases the perceived value of attaining the goal, while the “negative reality” increases the perceived effort required to attain the goal. In line with this explanation is the finding that subjects who only complete the “positive fantasy” component of the procedure become *less* motivated to attain the goal (98). Though this might seem initially surprising, it is easily explained by noting that the positive fantasy on its own will sharply *decrease* the discrepancy attributed to the goal, as subjects imagine the goal to already be completed; it is this decrease in discrepancy that demotivates these subjects.^27^ This is why the “negative reality” contrast, which counteracts the adverse effects of the “positive fantasy” component on discrepancy while maintaining its positive effects on value, is necessary for the mental contrasting procedure to work.

Thus the mental contrasting procedure is well-designed to increase the activation of a consciously chosen goal. So, given our characterization of volitional self-control, we should expect the mental contrasting procedure to help agents overcome temptation by incentive salience desires. And this is what we find. Oettingen et al. (89) found that the mental contrasting intervention caused smokers who wanted to quit to take more immediate action toward quitting than subjects who underwent a control intervention. And for non-addicted subjects, Johannessen et al. (99) found that dieters who performed the mental contrasting procedure were significantly more successful than control subjects at reducing their caloric intake over a 2-week period.

We have portrayed volitional self-control as involving a competition between the self-control and incentive salience systems over the activation of goals. We take this picture to be nicely confirmed by the fact that the mental contrasting procedure, which increases the activation of deliberatively chosen goals, helps agents to overcome temptation by both addictive and non-addictive incentive salience desires. Mental contrasting helps agents succeed in *motivating* themselves to act in accordance with their deliberative judgment – which, as we have seen, is not a trivial task.

### Implemental stage

#### Locus of implemental self-control conflict: habits

As we have said, a goal, once activated, will automatically guide behavior toward its own fulfillment. Thus, one might think that choosing the right goal in the face of temptation is sufficient for controlling one’s behavior. However, goal implementation – the process of executing one’s chosen goal pursuit in action – itself poses non-trivial self-control challenges.

This is because goals are not the only mental states that directly influence behavior. There are also *habits*, which Neal et al. (100) define as “response dispositions that are activated automatically by the context cues that co-occurred with responses during past performance” (198). In other words, habits are associations between contexts and behaviors that lead agents to produce a certain behavior when they encounter a certain contextual cue.

For our purposes, it is important to distinguish habits both from goals and from incentive salience desires. The distinction between habits and goals is essential to understanding the difference between the volitional and implemental stages of self-control. And as we emphasized earlier (see Desire), the habits that are produced by addiction are an importantly different phenomenon from the incentive salience desires that produce addiction. Habits and incentive salience desires may each exert their influence in the absence of the other, though they often go hand in hand.

The primary feature that distinguishes habits from goals is their *motivation-independence*. As habits are associative states that produce a behavior directly when a certain context is encountered, they do not depend for their influence on any motivation to engage in the relevant behavior. This is in contrast with goals, which are almost always activated by and dependent upon a desire to achieve some end.^28^ When one ceases to desire the end of a certain goal pursuit, the goal itself is deactivated (101); in contrast, when one ceases to desire the end that is served by a certain habit, the habit remains (102). One might, for instance, habitually make a turn that follows the well-worn driving route to one’s workplace, when in fact one does not want to go there at all, but rather is going to a restaurant that is actually in the opposite direction. However, one will never set out to pursue the goal of going to one’s workplace when in fact one has no desire whatsoever to do so.

The primary feature that distinguishes habits from incentive salience desires is their *motivational neutrality*. In addition to exerting their influence independently from (and even contrary to) one’s prior motives, habits also do not *produce* any desire to perform the habitual behavior. In other words, one does not *crave* acting out one’s habits. Schroeder and Arpaly (53) make this point well:

When one does not do something one wanted to do, there is often a little disappointment or regret. But when one does not make a habitual left turn, there is no disappointment or regret that coincides with not acting out of habit … [one] neither longingly thinks of making the left turn when at other intersections, nor is behaviorally disposed to get into a position to make the left turn. The habit only has influence upon behavior (231).

This apt observation about the different phenomenologies of habit and desire is confirmed by empirical research. As we have already mentioned (see Desire), simply learning to notice a habitual behavior seems to be sufficient for ceasing it, implying that once the subject becomes aware of the habitual behavior, it takes little additional self-control to override it (15, 16). Contrast this with incentive salience desires, which are still quite difficult to override even when one is reflectively aware of them.

A third feature of habits distinguishes them from both goals and incentive salience desires: their *behavioral inflexibility*. Neal and Wood (103) observe that “people rarely substitute habitual behaviors (e.g., a habit of daily jogging) for alternative behaviors that meet the same ostensible goal (e.g., switching from jogging to cycling)” [Ref. (103), p. 449]. We think this observation reflects an important fact about the structure of habits: they are associations of contexts with *a particular behavior*, not with an end that can be brought about by many different behaviors. Habits rigidly produce a certain behavior, never switching to producing a different behavior that better facilitates some goal. This is illustrated by a study on habitual popcorn eating in the cinema, in which subjects ceased to habitually eat popcorn if they were forced to do so with their non-dominant hand (102). This result shows that these subjects’ habit was not really *to eat popcorn*, but rather *to scoop popcorn into their mouths using their dominant hand*. When this behavior was no longer possible, the habit did not cause the subjects to engage in the alternative behavior of eating with their non-dominant hands – because *that* is not the particular behavior they associate with the context of the cinema. In contrast, both goals and incentive salience desires are very flexible in the behaviors they produce, dynamically switching between behavioral routines when doing so is adaptive for achieving their end (104, 105).

In summary, habits are best understood as a brute, direct association between a specific context and a rigid behavior, which produces behavior in a way that is unmediated by desire. This distinguishes habits from both goals and incentive salience desires, allowing us to see the task of controlling one’s habits as distinct from the task of controlling one’s goals. As it arises in the implementation of one’s goals, we will call this task the *implemental stage* of self-control.

#### Role of the incentive salience and self-control systems in creating habits

As Aristotle observed [Ref. (106), *Nicomachean Ethics* 1103a–b] and contemporary research has confirmed (107), habits are created by repetition. More precisely, a habit to perform a certain behavior in a certain context is created by an agent’s performing that particular behavior in that particular context many times before. This repetition ingrains the automatic association between context and behavior that constitutes the habit.

Both the incentive salience and self-control systems can create and sustain habits by this simple method. If an incentive salience desire is served by regularly performing the same behavior in the same context (say, ordering your usual beer at your favorite bar, or reaching for the ice cream in your freezer upon arriving at home), then by repeatedly acting on that incentive salience desire, one may create a habit that serves the desire. Insofar as one disapproves of the incentive salience desire, these may be called “bad habits.” Addicts, who usually spend a good while acting on their addictive desire before seeking help, will thereby acquire many habits that facilitate their addictive behavior. These “bad habits” will remain even when the addict has overcome her desire for the addictive substance, and may make it more difficult for the addict to remain in control, as Schroeder and Arpaly point out [Ref. (53), p. 228].

On the other hand, one may also inculcate “good habits” by repeatedly performing a behavior in a context that facilitates one of the cognitive desires or values on the basis of which one exerts self-control. For instance, one might create a habit of walking to the gym immediately after leaving work by simply exerting the self-control required to do so deliberately every day, until it becomes automatic and effortless. Many other examples of the self-controlled creation of habits come from athletics, music, and other skilled behaviors, where one exerts a great deal of self-control to repeat a certain behavior in a precise way during practice (whether a scale on the violin or a free-throw in basketball) and then, as one becomes skilled, is able to do the same behavior automatically and habitually. This self-controlled formation of “good habits” works just the same way as the formation of “bad habits” by the incentive salience system: produce the same behavior in the same context over and over again, and *voila!* – a habit is born.

#### How to improve implemental self-control: implementation intentions

Implemental self-control becomes a challenge when one has a *good goal* that may be thwarted by a *bad habit*. In other words, even once you have succeeded at *volitional* self-control, activating a goal that accords with your cognitive desires, your pursuit of this goal may be hampered by habits that lead to goal-discrepant behaviors. This problem will be especially dire if, as in the case of addicts, one’s goal is to change one’s behavior from a longstanding pattern produced by the pursuit of a powerful incentive salience desire. As Schroeder and Arpaly observe, bad habits may tip the balance in the addict’s self-control conflict, as when an addict finds herself habitually putting herself in situations that make drugs available or tempting.

One strategy for implemental self-control is simply to directly override the habit once it has been triggered. Though this works, it is difficult, causing ego depletion in ordinary subjects (28, 32). Overriding a habit is difficult not necessarily because it is difficult to overcome a habit once it has been detected, but because it requires a great deal of attention regulation to constantly monitor for the cues that trigger the habitual behavior. Given the limitations of our resources for self-controlled attention, this strategy for overcoming bad habits is itself quite limited.

An implemental self-control strategy that may escape these limits is suggested by research on *implementation intentions*, a technique created and investigated by Peter Gollwitzer. Implementation intentions are plans of the form “*if* I encounter X cue, *then* I will perform Y response!” Subjects who form implementation intentions to aid them in a goal pursuit have been shown in a large number of studies to pursue their goals much more effectively than subjects who simply form goal intentions (of the simpler form “I will do X!”). A meta-analysis of 94 studies involving over 8,000 participants found that the improvement of goal pursuit by implementation intentions over mere goal intentions is highly statistically significant, and medium-to-large in effect size [Cohen’s *d* = 0.65; (108)].

The helpful effects of implementation intentions seem to be largely due to the automatic association such intentions create between the “if” cue and the “then” response. Subjects who form implementation intentions afterward show a strong automatic association between the “if” cue and the “then” response, reacting far more quickly than controls to words associated with the “then” response after being primed with the “if” cue (109–112). This association leads subjects to quickly and automatically execute the intended “then” response when they encounter the specified “if” cue. The automaticity of this process explains why implementation intentions are just as effective (and in some cases *more* effective) when subjects suffer from impairments in executive control caused by cognitive load (113, 114), ego depletion (115), drug withdrawal (113), schizophrenia (113), ADHD (116, 117), or old age (118). The automaticity of implementation intentions is also indicated by studies showing that subjects will execute the “then” response of their implementation intentions even when the “if” cue is presented subliminally (119, 120).

The attentive reader will have already noticed that the kind of state created by implementation intentions – an automatic association between a cue and a response – is one and the same as the kind of state we have identified with *habits*. This implies that implementation intentions can enable an agent to deliberately create *new* cue-response associations that can compete with and override her old cue-response associations, i.e., her habits. If this is correct, then implementation intentions may provide a powerful tool for overriding unwanted habits and thus improving implemental self-control.

The research has borne this hypothesis out: subjects who form implementation intentions are significantly more successful at creating new habits and overriding old habits than control subjects who form mere goal intentions to do so (112, 121–125). As we would expect, reaction-time tasks indicate that implementation intentions break habits by creating a new association between the cue and the intended “then” response, which competes with the old association between the cue and the habitual response. After forming an implementation intention to break a habit, subjects react equally quickly to words associated with the intended “then” response as they do to words associated with the habitual response, indicating that the implementation intention levels the associative playing field (112). As the experimenters themselves put it: “implementation intentions eliminated the cognitive advantage of the habitual means in the ‘horse race’ with the alternative response” [(112), p. 503]. This gives the agent’s self-control system a much better chance of winning the larger “horse race” with the incentive salience system for the control of behavior.

We should thus predict that forming implementation intentions should help agents to overcome incentive salience temptation; and the available data support this prediction. With regards to non-addicted subjects, many studies have shown implementation intentions to significantly improve success in *dieting*, an activity that requires overcoming incentive salience desires for unhealthy foods (126–128). Regarding the effectiveness of implementation intentions in overcoming addiction, there is an unfortunate dearth of research. However, one study has found that forming implementation intentions helped adolescents to quit smoking, though only for those who had a “weak or moderate” smoking habit as measured by a standard scale (125).

It is important to note that since implementation intentions aid specifically with implemental self-control, they will only facilitate self-control success among subjects who have *already* succeeded in overcoming their incentive salience desires in both the deliberative and volitional stages of self-control. If self-control fails in either of these prior stages, then the deck will be stacked too heavily in favor of the incentive salience system for a purely implemental intervention such as forming implementation intentions to make much of a difference. Perhaps this is why implementation intentions on their own did not affect the most addicted subjects’ success at quitting smoking.

More generally, since success at all three stages of self-control is required for an agent to fully overcome incentive salience temptation, the most effective interventions to aid self-control will involve a combination of the stage-selective interventions we have advocated here. One existing intervention that follows this prescription is Gollwitzer and Oettingen’s “Mental Contrasting with Implementation Intentions (MCII) ” method, in which subjects first undergo the mental contrasting procedure – thus facilitating volitional self-control – and then form implementation intentions – thus improving implemental self-control. It should be no surprise that the MCII method is highly effective in aiding subjects to achieve their goals (117, 129–132). We can speculate that combining mindfulness training with the MCII method would augment self-control even further, comprising a “triple threat” of interventions that improve self-control in the deliberative, volitional, and implemental stages. Whether or not this “MMMCII” method (Mindfulness Meditation, Mental Contrasting, and Implementation Intentions) would in fact be effective in overcoming both addictive and non-addictive temptation is a question for further empirical work.

## Conclusion

Intentional action is the product of a competition between at least three different motivators, incentive salience desires, cognitive desires, and habits, which is mediated by the self-control system. As we argued in “Desire,” the incentive salience system is not only the source of addictive desires, but is the source of many of our ordinary, non-addictive desires as well. Due to the associative manner in which they are formed, these incentive salience desires are stubbornly independent of an agent’s reflective judgments about what is valuable. This gives rise to the problem of self-control: the challenge of resisting one’s incentive salience desires when they do not align with one’s cognitive desires. We argued in “The Existence of Self-Control” that the capacity to exert self-control plays an independent role in determining behavior over and above the relative strengths of an agent’s desires. This fact is illustrated most vividly by cases where the capacity to exert self-control is impaired (as in ego depletion) or lost altogether (as in vmPFC lesioning). The empirical evidence thus lends significant credence to the Platonic idea that there are two parts of the soul, one rational and the other appetitive, that compete for control over action.

As we argued in “Three Stages of Self-Control,” this competition proceeds in stages. We distinguished three of these stages: deliberative, volitional, and implemental. In the deliberative stage, an agent forms a judgment as to what course of action would be best. Since the judgment the agent reaches depends upon the considerations she attends to when deliberating about what to do, deliberative self-control is a matter of directing *attention* in order to resist the biasing pull of craving. In the volitional stage, an agent forms an intention to act in accordance with her deliberative judgment. What this amounts to is the activation of a *goal*, a mental state that guides behavior toward the achievement of a certain end. Since incentive salience desires automatically activate goals regardless of whether the agent judges them good, an agent must exert self-control in order to make her goals accord with her evaluative judgments. Finally, in the implemental stage, an agent must guide her behavior in pursuit of her chosen goal. Whether she succeeds in doing so depends upon her habits – the automatic associations between contexts and behaviors she has formed in the past. Since habits guide behavior independently from goals, the regulation of *habits* – both by overcoming bad habits and by forming good ones – is a third task of self-control, separate from the two preceding. An agent must succeed in all three of these stages of self-control in order to conform her actions to her cognitive desires.

This single model captures the predicaments of the addict and non-addict alike. The incentive salience desires that render the addict’s actions so wildly out of sync with her values are present in non-addicts as well, though in less extreme form. And thus the non-addict will also sometimes act in ways she does not endorse, driven by desires that motivate independently of her conception of the good. The non-addict can resist these desires by exerting self-control; but the addict can do this too. The task of self-control is far more difficult for the addict – which is why it is often unreasonable to blame addicts for giving in to temptation even when we might blame a non-addict for doing so. But self-control is possible for addicts, especially with strong incentives and assistance from others. Indeed, this is just what recovery from addiction is: the addictive desire does not go away, but the recovering addict learns to control her behavior in spite of it.

Thus addicts are not so different from the rest of us as we may have thought. But that may be because we underestimated our own similarity to addicts, rather than the other way around. There is a tendency to think of human agency as an entirely rational affair: we simply do whatever we think is most likely to get us what we want. The heuristics and biases literature has undermined this picture somewhat over the past few decades, but only by showing us how we are not always rational in selecting the *means* to our ends (133, 134). The model we have defended here shows that the irrationality – or arationality – of human agency goes a step deeper: our ends themselves can be set by desires that are utterly divorced from what we take to be rationally desirable. The activity of controlling our actions is thus not merely a matter of figuring out what we ought to do; it is a matter of fighting to control our minds and actions in accordance with our reasons. To borrow Plato’s metaphor, being a human agent is more like struggling with stubborn horses for control over a chariot than it is like calculating a utility function. Those of us who are lucky enough not to suffer from addiction might come to understand ourselves better by acknowledging that there is an addict in us all.^29^

## Conflict of Interest Statement

The authors declare that the research was conducted in the absence of any commercial or financial relationships that could be construed as a potential conflict of interest.

## Footnotes

^1^We do not take our account to provide an exhaustive explanation of addiction (or ordinary temptation, for that matter). It leaves out at least two important factors: affect and social context. Affect comes into the explanation of addiction in two places: first, one of the major reasons why addicts use is in order to relieve negative emotions such as stress, anxiety, and depression (135); and second, these negative emotions may significantly impair agents’ ability to exert self-control (136). And as has been recognized since the earliest addiction treatment programs, social context plays a huge role in addiction: social influence often explains why the addict started taking the drug in the first place; and social support is an essential ingredient in the process of recovery from addiction. Our account has little to say about these important phenomena. Rather than attempting to survey all of the explanatory factors in addiction, we aim only to characterize two of these factors – desire and self-control – and the interactions between them. (Thanks to both Serge Ahmed and Hanna Pickard for pointing out these limitations of our account).

^2^See Batson and Shaw (137) for a classic empirical argument for this claim.

^3^The particular interpretation here follows that given in Holton and Berridge (9); readers should look there for much more detail on what is here treated far too swiftly.

^4^Holton and Berridge (9), in an attempt to avoid prejudicing the case, called these “A-signals” and “B-signals.” We have replaced this terminology with something a little more memorable.

^5^Although there remains some controversy here, it looks as though the formation of beliefs can take place in the absence of dopamine (for instance, in genetically modified dopamine deficient mice) and hence in the absence of motivation. See Robinson et al. (138); and for a recent discussion of the competing hypotheses together with a proposal for compromise, see Berridge and O’Doherty (139).

^6^Indeed, withdrawal, horrible though it can be, plays a minor role in addiction; consumption is not primarily motivated by a desire to avoid it.

^7^We say “can,” for it remains an open question why most people who consume addictive drugs do not become addicted to them (most people in the West drink alcohol, but most do not become alcoholics); and likewise why most animals do not consume addictive drugs if they have attractive alternatives (see below). It could be that they are less prone to form the relevant incentive salience desires; it could be that they form competing desires more strongly; or it could be that they are better at self control. For a survey of the mounting evidence for the former, see Saunders and Robinson (140).

^8^For a review of the evidence that there is more going on in the formations of desires for sugar than simply the activity of the dopamine system see DiLeone et al. (141) and Ahmed et al. (142).

^9^Such an approach has been advocated, in rather different ways, by Scanlon (143) and Railton (144). We agree that some desires have this feature, but deny that this is the only kind of desire

^10^It is very effective.

^11^We do not take this distinction to be exhaustive. There could be desires that are not cognitive, in the sense that they are not sensitive to our judgments about reasons, but are not incentive salience desires either, since they are not produced by the incentive salience system. The desires involved in emotional reactions such as fear or guilt, for example, do not seem to fall neatly into either category.

^12^See [(6), pp. 112–136].

^13^This last qualification is necessary since there is some evidence that helping those with whom one does empathize can be rewarding in itself. In general, our argument applies only to moral behavior that is not intrinsically pleasurable; and quite where the boundaries of that lie is not yet clear.

^14^It is important to emphasize that we do not take this characterization to apply generally to addicts, but only to persons driven *exclusively* by incentive salience desires; as we argue later in this section, it is implausible to think that this is true of most addicts. Thanks to Hanna Pickard for pressing us to clarify this point.

^15^For more evidence beyond the IGT supporting the idea that vmPFC lesion patients are insensitive to long-term consequences, see [Refs. (145, 146)].

^16^To return to an earlier point: we are not claiming that this is the *only* deficit that occurs in vmPFC lesion patients. Naturally occurring brain lesions are messy by nature and will rarely selectively impair a single psychological process without disrupting others. For instance, vmPFC lesion patients’ reported abnormalities in moral judgment (147), social cognition (148), and affective experience (23) are not straightforwardly explained by our hypothesis that they suffer from impaired self-control. However, we think our hypothesis provides a better explanation for vmPFC lesion patients’ deficits in social behavior and long-term planning than the emotion-based explanation given by Damasio (22), though we do not have the space to argue this point here.

^17^Levy (7) offers a similar argument for the existence of the self-control system, though he takes it to be part of System 2. However, Levy’s view differs significantly from ours, in that he takes the self-control system to play an exclusively cognitive role: “ego depletion is caused by engagement in any of the much broader class of system 2 processes, which involve effortful cognitive processing” [(7), p. 147]. He argues that self-control’s role in overcoming temptation is fully mediated by its role in forming accurate, unbiased beliefs about what is best to do [see also (51, 52)]. While we agree that this is *one* of the functions of self-control (see Deliberative stage), we differ from Levy in ascribing to the self-control system some directly motivational functions as well. A result of this difference is that Levy’s picture omits the very role of the self-control system we have most emphasized: namely, its function of overriding an agent’s incentive salience desires to control behavior in accordance with her cognitive desires. So, though Levy’s argument is similar to ours, it does not suffice for the point we wish to make here.

^18^Thanks to Serge Ahmed and Bennett Foddy for both raising this worry.

^19^See Holton and Berridge (9) for discussion of this.

^20^Henden (149) argues that compulsive actions (which he takes addictive actions to be) are caused by habits: “an action is compulsive, not because it is caused by an irresistible desire, but because it is part of a habit the compulsive person would find extremely difficult to discontinue even if she made a sincere effort to do so” (363). He would thus seem to be another advocate of the view that the locus of self-control conflict lies in the regulation of habits. However, Henden uses “habit” in a much wider sense than we do: he defines “habits” as “behavior patterns regularly performed in characteristic circumstances” (371). In contrast, we are using “habit” to refer to a particular psychological state, distinct from desires, goals, or intentions, that involves a strong and rigid association between a contextual cue and a behavior that causes the behavior to be automatically triggered by the cue (see Implemental stage for more detail). Thus “habits” in Henden’s sense may or may not be caused by “habits” in our sense. In fact, a closer reading of Henden reveals that the “habits” he takes to underlie addiction are caused, not by the rigid stimulus-response associations we are calling “habits,” but rather by incentive salience desires, which Henden calls “inclinations.” Thus we do not take Henden to advocate the view that the locus of self-control is the regulation of habits, in the sense we are using the term. Thanks to Hanna Pickard for drawing our attention to this connection with Henden’s work.

^21^The stages we propose are inspired by Peter Gollwitzer’s highly influential *Rubicon model of action phases* (150). Though our division of stages does not correspond exactly with Gollwitzer’s, we doubt this reveals a substantive disagreement, but rather reflects a difference in focus. Along similar lines, our stages are not the same as Rorty’s proposed stages, but we think this is only because Rorty makes more fine-grained distinctions between stages than we do. Though we have limited ourselves to only those distinctions between stages for which we have empirical evidence, we are open to the possibility that there may be more useful distinctions between stages than we have made here.

^22^It is important to note that these stages are *goal-relative*: an agent might be in one stage relative to one goal while in a different stage relative to another. For example, an agent may have decided to take a trip to New York; having formed this intention, she is now in the implemental stage of this goal pursuit. However, in the process of implementing her intention, she will need to deliberate about further matters: should she take the train or a plane? Thus she might be in the deliberative stage regarding the question of *how to get to* New York even while she is in the implemental stage regarding her intention to *go* to New York. So the question to ask is not: what stage of self-control is this agent in *full stop*; but rather: what stage of self-control is this agent in *for this particular goal pursuit*?

^23^Subjects who chose the extra dose would receive two doses of BUP rather than one on the appointed day. This was a significant incentive: “Although a single dose of BUP is sufficient to eliminate addicts’ acute cravings, a double dose produces a longer, more satisfying high” [(60), p. 869].

^24^It might also be the case that evaluative judgments are formed *subsequently* to the intentions: in the light of what an agent has decided to do, cognitive dissonance motivations might lead her to form judgments that present those decisions in a good light. But we still contend that intentions and judgments have genuine independent existence [see Ref. (6), pp. 1–19].

^25^We are thus using the term “goal” to refer not to the state of affairs one is pursuing (as “goal” does when used colloquially, e.g. “my goal is to lose 5 pounds”), but rather to the mental state that guides one’s behavior towards bringing about that state of affairs.

^26^This claim might seem counterintuitive, if we think of goals on the model of desires. If I want to go to the opera *and* want to go to the movies, but only get to pick one, I will still desire the other: at the movies, I’ll worry about what I’m missing at the opera, or vice versa. But goals are *not* like desires; they are like *intentions*. And though it is common to have conflicting desires, it is difficult to maintain conflicting intentions. Though it is possible for agents to have incompatible intentions, there is pressure, both rational and psychological, to maintain consistency among one’s intentions. (Compare belief: though it is possible to have inconsistent beliefs, there is pressure, both rational and psychological, to resolve any such inconsistencies once they come to light). When I form the intention to go to the opera, my attention and behavior are directed to the pursuit of *that* end, and thereby away from the end of going to the movies. I look up the time of the opera, buy tickets, and get directions to the opera house; I *don’t* look up the movie times, buy movie tickets, and get directions to the movie theatre. This tendency for goals to narrow an agent’s focus, excluding incompatible courses of action, is what we have in mind when we say that “only one goal can guide behavior at a time.” Thanks to Hanna Pickard for raising this concern.

^27^In fact, the act of imagining goal completion has been shown in one study to lead to “goal turnoff,” the suppression of goal accessibility that usually occurs after the goal has *actually* been completed (151).

^28^A possible exception to this claim is the case of unconscious goal priming by exposure to words semantically associated with a goal (104).

^29^For helpful comments and criticism, we thank Serge Ahmed, Dylan Bianchi, Bennett Foddy, Matthias Jenny, Hanna Pickard, Bernhard Salow, Ian Wells, and the participants at the Mechanisms of Self-Control Workshop at King’s College London.
